# Dietary Inflammatory Potential in Pediatric Diseases: A Narrative Review

**DOI:** 10.3390/nu15245095

**Published:** 2023-12-13

**Authors:** Martina Tosi, Chiara Montanari, Federica Bona, Chiara Tricella, Marta Agostinelli, Jonabell Dolor, Claudia Chillemi, Elisabetta Di Profio, Veronica Maria Tagi, Sara Vizzuso, Giulia Fiore, Gianvincenzo Zuccotti, Elvira Verduci

**Affiliations:** 1 Department of Pediatrics, Vittore Buzzi Children’s Hospital, University of Milan, 20154 Milan, Italy; martina.tosi@unimi.it (M.T.); chiara.montanari@unimi.it (C.M.); federica.bona@unimi.it (F.B.); chiara.tricella@unimi.it (C.T.); marta.agostinelli@unimi.it (M.A.); jonabell.dolor@unimi.it (J.D.); claudia.chillemi@unimi.it (C.C.); elisabetta.diprofio@unimi.it (E.D.P.); veronica.tagi@unimi.it (V.M.T.); sara.vizzuso@asst-fbf-sacco.it (S.V.); gianvincenzo.zuccotti@unimi.it (G.Z.); 2 Department of Health Sciences, University of Milan, 20146 Milan, Italy; elvira.verduci@unimi.it; 3 Department of Biomedical and Clinical Science, University of Milan, 20157 Milan, Italy; 4 Metabolic Diseases Unit, Department of Pediatrics, Vittore Buzzi Children’s Hospital, University of Milan, 20154 Milan, Italy

**Keywords:** inflammation, dietary inflammatory potential, ultra-processed food, Western diet, non-communicable diseases, metabolic diseases, gut dysbiosis

## Abstract

Inflammatory status is one of the main drivers in the development of non-communicable diseases (NCDs). Specific unhealthy dietary patterns and the growing consumption of ultra-processed foods (UPFs) may influence the inflammation process, which negatively modulates the gut microbiota and increases the risk of NCDs. Moreover, several chronic health conditions require special long-term dietary treatment, characterized by altered ratios of the intake of nutrients or by the consumption of disease-specific foods. In this narrative review, we aimed to collect the latest evidence on the pro-inflammatory potential of dietary patterns, foods, and nutrients in children affected by multifactorial diseases but also on the dietetic approaches used as treatment for specific diseases. Considering multifactorial diet-related diseases, the triggering effect of pro-inflammatory diets has been addressed for metabolic syndrome and inflammatory bowel diseases, and the latter for adults only. Future research is required on multiple sclerosis, type 1 diabetes, and pediatric cancer, in which the role of inflammation is emerging. For diseases requiring special diets, the role of single or multiple foods, possibly associated with inflammation, was assessed, but more studies are needed. The evidence collected highlighted the need for health professionals to consider the entire dietary pattern, providing balanced and healthy diets not only to permit the metabolic control of the disease itself, but also to prevent the development of NCDs in adolescence and adulthood. Personalized nutritional approaches, in close collaboration between the hospital, country, and families, must always be promoted together with the development of new methods for the assessment of pro-inflammatory dietary habits in pediatric age and the implementation of telemedicine.

## 1. Introduction

Inflammation is well known to be a crucial component of the pathophysiology of several chronic diseases [1]. The growing awareness that dietary habits play an essential role in the development of a chronic inflammatory status has led to a particular focus of the research in the field of pro-inflammatory diets, in order to limit long-term complications [2]. Different nutritional compounds, including micro- and macronutrients, bioactive molecules, and specific dietary patterns, can influence the inflammation process [3]. Anti-inflammatory dietary patterns in the adult population usually imply a significant consumption of vegetables, fruits, whole grains, a moderate intake of legumes and fish, and a low consumption of red meat. They are usually rich in polyunsaturated fatty acids (PUFAs), particularly marine *n*-3 PUFAs, vitamin C, vitamin E, carotenoids, and polyphenols [4,5,6]. The mediterranean diet (MD) is an example of an anti-inflammatory dietary pattern [7]. The current evidence highlights that the MD is associated with lower levels of inflammatory biomarkers, particularly C reactive protein (CRP) and interleukin-6 (IL-6) [8,9]. In contrast, the Western diet (WD), consisting of a high caloric intake and a frequent consumption of sugars, refined cereals, red and processed meats, has been linked with an increased pro-inflammatory potential and higher levels of CRP and IL-6 [10]. The WD includes oxidized lipids, saturated fatty acids (SFAs), trans fatty acids, food additives, and ultra-processed foods (UPFs) [3]. Over the last few decades, the incidence of intestinal and systemic inflammatory disorders has increased, particularly among children and adolescents. This result might also be driven by the increasing popularity of the WD [11]. 

**Figure 1:**
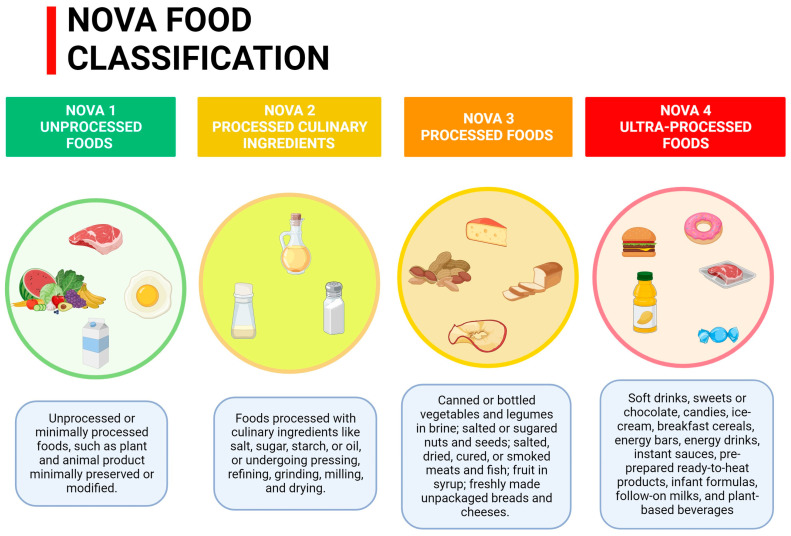
NOVA Food Classification.

UPFs, according to the NOVA food classification system (Figure 1), are readily available meals that are often used as a practical substitute for traditional ones [12,13,14]. They are characterized by higher concentrations of sugars, SFAs, and sodium [15]. Moreover, these processed foods are energy dense, highly palatable, and impact the glycemic load. They are also lower in protein, dietary fiber, micronutrients, and phytochemicals, compared to their counterparts [16]. 

Apart from an altered nutritional profile [16,17,18,19,20,21,22,23,24,25], UPFs can also be rich in additives and emulsifiers (e.g., carrageenan) implicated in the inflammatory cascade [26,27] or food packaging contaminants, such as bisphenol and phthalates, known for their potential role as endocrine disruptors [28,29]. In addition, it has been hypothesized that these substances may negatively modulate gut microbiota composition [30,31]. 

The association between UPFs’ consumption and the pathogenesis of inflammatory diseases has been established in adults, and some evidence suggests their pro-inflammatory effect in children, who are usually the most frequent UPFs consumers, especially during school age [32,33,34]. At present, the striking question is about understanding to what extent the adoption of a pro-inflammatory dietary pattern could influence the onset of NCDs, such as obesity, metabolic syndrome, diabetes, and inflammatory bowel disease (IBD) in pediatric age and onwards, but also allergies, cancer, and celiac disease.

At the same time, it is interesting to point out that some pathological conditions, including celiac disease and inherited metabolic diseases (IEMs), require lifelong special diets, excluding some determined nutrients or compounds or exceeding the consumption of others, which may alter the gut microbiota composition, promoting the overgrowth of bacteria-linked intestinal inflammation [35], and also cause a depletion of short-chain fatty acids (SCFAs)-producing bacteria. SCFAs, mainly represented by acetate, propionate, and butyrate [36], are products of microbial fermentation in the large bowel from food components that are unabsorbed/undigested in the small intestine. These compounds have been demonstrated to play anti-inflammatory roles, contributing to the maintenance of the gut barrier’s function and to the promotion of gut homeostasis [37,38]. 

In this narrative review we aim to examine the latest evidence on the pro-inflammatory potential of dietary patterns in children and adolescents affected by multifactorial diseases (diet-related, immuno-mediated, allergies and malignancies) but also on the dietetic approaches used as treatments for specific diseases (celiac disease or IEMs). 

## 2. Materials and Methods

**Figure 2:**
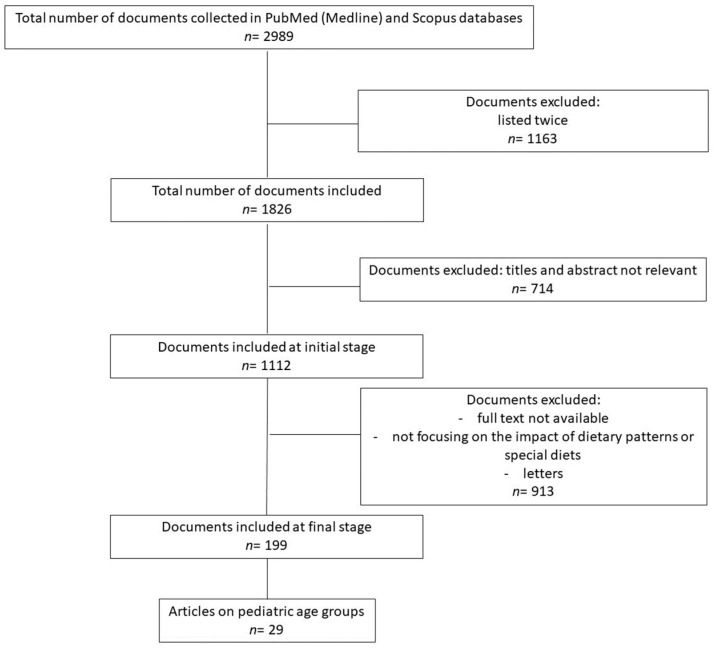
Flow chart of study selection.

The authors have independently searched, via the PubMed (Medline) and Scopus databases, for the most relevant articles published from 2008 to June 2023. The research strategies used are reported in Appendix A. The total number of documents collected in the different databases was *n* = 2989. The flow selection data are illustrated in Figure 2. 

Documents were excluded at different steps: when listed twice (*n* = 1163), or when their titles and abstract were not relevant (*n* = 714). Although numerous studies collected (*n* = 1112) had a connection between inflammation and pediatric diseases, the authors chose to focus the review only on papers regarding the impact of dietary patterns, special diets, specific foods, or nutrients, on the onset or progression of the disease. The most relevant studies, including original papers, meta-analyses, clinical trials, and observational studies, were selected. Letters were excluded. Articles on the adult population were added and used to expand the discussion. A final number of 199 articles were selected, of which 29 were on pediatric age groups. Preclinical studies were considered only to complement descriptions of the pro-inflammatory diets’ mechanisms of action, thereby unifying the molecular basis with the randomized controlled trials’ (RCTs) evidence. Of the IEMs, only Phenylketonuria (PKU) and glycogen storage diseases (GSDs) were included, as enough publications ware available.

## 3. Results

The pediatric diseases presented were categorized in two main groups: multifactorial diseases, in which dietary patterns and nutrition might be involved in the pathogenetic mechanisms; and diseases that require a special diet as the main treatment. For the purpose of the present manuscript, IBD and multiple sclerosis (MS) have been classified as being in the first group, although they also share features with the second group.

### 3.1. Dietary Inflammatory Potential as a Trigger in Multifactorial Diseases

#### 3.1.1. Diet-Related Diseases 

##### Obesity and Metabolic Syndrome

Obesity is an increasing global health problem, both in children and adults, associated with several comorbidities, including metabolic syndrome (MetS).

MetS is a cluster of metabolic abnormalities that includes central obesity, dyslipidemias (low HDL cholesterol and high triglyceride levels), hypertension, and insulin resistance. It is characterized by a chronic low-grade inflammation status and oxidative stress, and it is associated with a significant risk of developing type 2 diabetes and cardiovascular diseases [39]. Moreover, MetS can also show liver involvement that has been recently regarded as metabolic dysfunction-associated fatty liver disease (MAFLD) [40].

Diet is considered one of the major contributors to obesity. Recent studies have shown that the WD pattern, rich in SFAs and trans fats but low in omega-3 PUFAs, contributes to both insulin resistance and the increased secretion of inflammatory markers [41,42]. Additionally, children and adolescents adopting a “sweet dietary pattern”, rich in cakes, pastries, sweets, and soft drinks, are at higher risk for abdominal obesity, elevated blood pressure, and MetS [43], and sweets’ consumption is associated with unfavorable cardiometabolic health outcomes, including higher levels of triacylglycerol, Very-Low-Density Lipoprotein (VLDL) cholesterol, and higher levels on the insulin resistance index (HOMA-IR) [44,45]. In line with this, the high consumption of sugary drinks was estimated to increase, up to 5 times, the risk of developing MetS in the adolescent population [46]. 

The MD is actually considered a protective dietary pattern against obesity and MetS [47]. Velázquez-López et al. [48] demonstrated that a higher adherence to the MD is associated with a decrease in the prevalence of MetS (from 16% to 5%) and especially with a decrease in the glucose blood levels and BMI of children and adolescents with obesity. Vegetables are known to be enriched in antioxidants such as flavonoids, carotenoids, tocopherols, and ascorbic acids, well-known players in lowering chronic inflammation. Instead, the WD replaces the intake of fresh or minimally processed foods, such as legumes, whole grains, vegetables, fruits, and oilseeds, all foods that are linked to a positive prevention against MetS and type 2 diabetes, with UPFs. Some studies, qualitatively evaluating the diet in both pediatric and adult age, have quantified UPFs’ intake and their concurrent association with obesity and MetS (see Table 1) [49,50,51]. Other components of UPFs, namely packing contact materials, such as bisphenol A and phthalates, may explain this association, since they are implicated in endocrine alterations and insulin resistance [52]. Recently, the effect of unbalanced diets on the gut microbiota has emerged; specifically, high intakes of fats and refined carbohydrates can lead to the selection of pro-inflammatory bacteria [53]. In a clinical study, Ramne et al. [54] illustrated that the consumption of added sugars and sugary drinks promotes an increased Firmicutes/Bacteroidetes ratio, while simultaneously displaying a negative correlation with the concentration of the Lacnhnobacterium genus, known for its beneficial role as a butyrate producer. 

The WD is also characterized by a deficient intake of micronutrients, usually found in children with obesity [55]. Some studies have shown an inadequate zinc intake and its consequent deficiency in children with overweight or obesity [56,57]. Furthermore, Ortega et al. [58] and Garcia et al. [59] reported that lower zinc levels in the pediatric population were associated with increased insulin resistance. At the same time, zinc demonstrated potential anti-inflammatory effects through cytokine signaling pathways and the reduction of plasma levels of IL-6, TNF-α, and CRP [55,60], with a protective effect against chronic low-grade inflammation, which is found in obesity and MetS.

Nutrients could influence the inflammatory pathways with a positive or negative effect, interacting with extracellular receptors and mediating intracellular signaling [55,61,62,63]. The MD, rich in nutrients and phytochemicals known for their anti-inflammatory role, including vitamins C and E, epigallocatechin gallate, lycopene, and polyphenols, could modulate inflammatory components such as NF-KB, mitogen-activated protein kinases, and IL-1β signaling [55]. Lastly, the high fiber content of the MD could have an anti-inflammatory role due to the major production of SCFAs by gut bacteria, which could improve glucose and lipid metabolism in many tissues [64,65]. The changes in the gut microbiota are strictly related to the type of fatty acids ingested: the altered *n*-6/*n*-3 PUFAs ratio was found to promote the development of Enterobacteriaceae and Clostridia spp., leading to a pro-inflammatory environment that could be attenuated by the introduction of *n*-3 PUFAs [66,67]. The major findings from observational and interventional studies concerning pediatric obesity are reported in Table 1.

**Table 1:** Summary of findings from observational and interventional studies concerning pediatric obesity.

Exposure: Dietary Patterns/ Nutritional Compounds	Authors (Year of Publication), Study Design	Population	Exposition/Outcome/Results	References
Healthy dietary pattern Sweet dietary pattern Western dietary pattern	Kelishadi, R. et al. (2018), matched case–control study design	3755 students (aged 7–18 years)	Sweet dietary pattern enhanced the risk of MetS, hypertension, and abdominal obesity.	[43]
Dietary habits assessed via a semi-quantitative FFQ for adolescents	Tavares, L.F. et al. (2012), cross-sectional study design	210 adolescents	High consumption of UPFs was associated with a higher prevalence of MetS (>1245 g/day of UPFs intake was associated with a 150% higher prevalence of MetS).	[50]
Zinc nutritional status (plasma, erythrocyte, and 24 h urine), Dietary habits assessed by 3 d food records	Cozzolino, S.M.F. et al. (2002), case–control study design	23 obese children and 21 controls (aged 7–14 years)	Zinc concentrations in plasma and erythrocytes were significantly lower in the obese group (diets consumed by both groups had marginal concentrations of zinc).	[57]
Dietary habits assessed by 3 d food records with special attention to zinc	Ortega, R.M. et al. (2012), cross-sectional study	357 schoolchildren (aged 8–13 years)	Children with Zn deficiency had higher HOMA-IR values.	[58]
Dietary habits assessed via FFQ and recalls, Adherence to the MD assessed by KIDMed score	George, E.S. et al. (2021), cross-sectional study	1972 (aged 9–13 years)	Poor adherence to the MD was associated with an increased likelihood for central obesity, hypertriglyceridemia, and insulin resistance.	[47]
Mediterranean diet vs. standard diet	Velasquez-Lopez L. et al. (2014), randomized controlled trial	50 children and adolescents (aged 3–18 years) treated with Mediterranean diet (60% of energy from carbohydrate, 25% from fat, and 15% from protein) (*n* = 24), or a standard diet (55% of carbohydrate, 30% from fat and 15% from protein)	The MD (16 weeks) improved the BMI, glucose, and lipid profile in children and adolescents with obesity and any MetS components.	[48]
Adherence to the Mediterranean diet	Mohammadi S. et al. (2022), cross-sectional study	203 adolescents	Higher adherence to the MD was related to lower odds of MUO.	[64]
High-fiber dietary pattern to test changes in inflammation indexes of the gut microbiota	Li H. et al. (2021), open-labelled and self-controlled study	Prader-Willi Syndrome (PWS) *n* = 18 (dietary intervention 30 days) and simple obese (SO) children *n* = 19 (dietary intervention 30 days)	In both cohorts, the high-fiber diet reduced the abundance of virulence factor, and particularly pathogen-specific, genes.	[65]

Abbreviations: Ultra-Processed Foods (UPFs); Food Frequency Questionnaire (FFQ); Mediterranean Diet (MD); Metabolic Unhealthy Obesity (MUO).

In conclusion, diet is a determinant in the development of obesity and MetS, and dietary patterns and nutrients have distinctive effects on the inflammatory response and metabolic compensation. Unbalanced diets, such as those rich in sugars, refined carbohydrates, SFAs and trans fatty acids, are associated with a pro-inflammatory status and should be avoided. Several studies discuss the benefits of nutrients with anti-inflammatory and antioxidant properties in modulating the low-grade inflammation triggered by obesity and metabolic syndrome, but further research is still needed.

##### Inflammatory Bowel Diseases 

IBDs, the main forms of which are ulcerative colitis (UC) and Crohn’s disease (CD), are characterized by chronic relapsing–remitting inflammation of the gastrointestinal tract. In these multifactorial diseases, diet plays the main role in the onset and progression of IBD by influencing the composition and functioning of the gut microbiota, intestinal barrier, immunity, and hormone release, acting on a genetic predisposition [3]. There is a close interconnection and mutual influence between the gut microbiota and host mucosal immune system: any slight disturbance in the microbial communities may contribute to intestinal immune disruption and, according to recent research, alterations of the gut microbiota may cause dysregulated mucosal immune responses, leading to the onset of IBD in genetically susceptible hosts [68,69].

Several studies have documented an increasing incidence of IBDs, especially as a result of the global spread of the Western lifestyle and eating habits [70,71,72]. In particular, its high intake of simple and refined carbohydrates promotes intestinal dysbiosis and inflammation [73]. An excess of any kind of carbohydrate in IBD patients with intestinal malabsorption may easily exacerbate existing intestinal dysbiosis, which in turn may contribute to the dysmetabolism of other nutrients [74]. 

On this basis, a dietary strategy proposed for patients with IBD is the Fermentable Oligosaccharides, Disaccharides, Monosaccharides And Polyols (FODMAP) exclusion diet, in order to relieve symptoms such as bloating, flatulence, cramping pain, and diarrhea, but more studies are necessary to assess the long-term effects and its safety profile on nutrient intake, as it is a restrictive diet [75,76]. Another proposed nutritional approach is the “specific carbohydrate diet” (SCD) which excludes gluten and restricts all carbohydrates except monosaccharides (glucose, fructose, and galactose), allowing fresh fruits and vegetables, with beneficial effects in controlling IBD although available data in adult and pediatric populations are still limited [77,78].

The possible interplay of specific dietary food components and the gut microbiota of IBD patients is shown in Table 2. 

Apart from fruit and vegetable intake [79,80] or meat consumption, known for their positive and negative implications, respectively [81,82,83], another point concerns the consumption of gastro-resistant proteins which are capable of modifying and exacerbating intestinal permeability [74], namely gluten [84,85,86] and caseins, from fermented and unfermented dairy products [87,88,89]. Therefore, dietary indications for IBD patients include a reduction in the overall amount of meat consumed, especially red and processed meat, and the elimination or significant reduction of gluten and dairy products (caseins), except for yogurt and kefir, which have shown positive effects [89].

In general, high-protein diets have a potential negative impact in patients with IBD, although the protein source of the diet is still debated [80]. In fact, the reduced intestinal ability to assimilate proteins, due to an intestinal disease, leads a portion of unabsorbed proteins reaching the colon, where they alter the composition of the gut microbiota by reducing beneficial bacteria such as Roseburia/Eubacterium rectale butyrate producers [74]. Moreover, an excess of protein can lead to an imbalance in the metabolites produced by colon bacteria catabolism (e.g., ammonium, hydrogen sulfide, p-cresol, and phenol), which can damage cells and the intestinal barrier [90]. 

A low fiber intake has been related to an increased risk of IBD: a meta-analysis on the association between fiber intake and the risk of IBD, including studies in children and the adolescent population, established a significant inverse relationship between a high fiber intake and risk of CD, but only a marginally significant association between fiber and a risk of UC [91]. Although excessive fiber consumption could exacerbate the gastrointestinal symptoms in these patients and may be contraindicated during the exacerbation phase of the disease, fiber intake is still recommended after remission by limiting the intake of insoluble fiber [77].

The WD is also characterized by a high intake of *n*-6 PUFAs and a low intake of *n*-3 PUFAs. Recent data address the protective effect of *n*-3 PUFAs in the prevention and therapy of UC, while the consumption of a higher ratio of *n*-6/*n*-3 PUFAs was associated with a higher incidence of UC [92]. The role of *n*-3 PUFAs in the prevention and therapy of CD still appears controversial [93]. The intake of *n*-3 PUFAs can reduce the production of pro-inflammatory agents (e.g., prostaglandin E2, thromboxane B2, and inflammatory mediators of hydroxyicosatetraenoic acid) and can increase the synthesis of molecules such as resolvins, protectins, and maresins, which counteract the dysbiosis related to IBDs and the down-regulation of pro-inflammatory genes. 

The global increasing incidence of IBDs also concurs with an increase in the consumption of UPFs [94,95,96]. A cohort study including 187.854 adults in the UK has shown that a high intake of UPFs has been associated with a significantly increased risk of CD but not UC [97]. Trakman et al. [98] demonstrated that Crohn’s disease patients, in early life (0–18 years), show an increased intake of UPFs compared to various control groups. 

UPFs are matter of concern due to both their high amount in salt [99] and the artificial sweeteners or maltodextrins often present in carbonated drinks, candies, and energy products [80,100], which can heighten the intestinal inflammation in IBD patients (see Table 2). Additionally, UPFs may contain nanoparticles such as titanium oxide and aluminum which could increase their susceptibility to colitis. An important role is played by the high content of emulsifiers, thickeners, and other additives in UPFs [101]. For example, emulsifiers, such as carboxymethylcellulose and polysorbate-80 [102], cause the disruption of the mucosal barrier and induce dysbiosis. Another commonly used additive is carrageenan, a gelling agent and thickener derived from algae, which induces inflammation in animal models: studies in animals show that carrageenan induces histopathological features similar to IBD, alters the gut microbiota, and disrupts the intestinal epithelial barrier. In some pilot studies in humans, the restriction of emulsifiers in diets has been associated with an improvement in CD-related symptoms [103].

In conclusion, diet appears to have a crucial role in the pathogenesis and clinical course of IBD, by modulating the inflammatory state underlying the disease and also through the modulation of the gut microbiota composition. The different dietary approaches above mentioned can optimize the management of these diseases; however, currently, the ideal dietary model is still debated. Clinical evidence of the role of the diet in IBD patients currently comes from studies conducted in adult populations, while evidence in pediatric populations is still lacking. 

#### 3.1.2. Immune-Mediated Diseases 

##### Type 1 Diabetes 

Type 1 diabetes (T1D) is one of the most common autoimmune diseases in childhood. In T1D, the immune response to self-antigens leads to the apoptosis of pancreatic β-cells, resulting in lower insulin production and hyperglycemia. The clinical management of T1D usually involves following a MD and carbohydrate counting [104]. As previously mentioned, this dietary pattern can affect the gut microbiota composition [105]. Intestinal dysbiosis, as a consequence, may play a role in the modulation of the immune system, and this mechanism is associated with an increased risk for allergic and autoimmune diseases [12]. A healthy diet, with a high intake of fiber and low levels of SFAs and sugars, can help to prevent gut dysbiosis. Thus, genetically predisposed children should be provided with a well-balanced diet to reduce the risk of developing T1D or other chronic diseases [12]. 

Recent publications describe how the consumption of UPFs may have a negative impact on the general health status [12] and the development of T1D in childhood, which seems to be associated with a high intake of sugars [106]. Aguayo-Patròn et al. [12] reported that a high intake of UPFs can lead to an increase in the susceptibility to autoimmune diseases such as T1D [12,107]. Until now, only Pang et al. [108] have conducted a study investigating the associations between UPFs’ consumption and obesity indicators among individuals with and without T1D. The authors reported that individuals with T1D may consume more UPFs than non-diabetic controls. Moreover, T1D participants who consumed the highest amount of UPFs had a higher risk of increasing their weight and BMI than those who consumed the least amount of UPFs over the 14 years of follow-up. However, no statistically significant associations were observed for waist circumference, overweight or obesity. A Swedish study reported that the higher intake of sugars, especially disaccharides and sucrose, is associated with an increased risk of T1D [106,109]. On the contrary, another study found that a high intake of carbohydrate-rich foods, but not of monosaccharides and disaccharides, increases the risk of T1D with a non-linear dose–response curve [110]. 

Lastly, regarding the introduction of specific foods and their association with T1D, the role of early nutrition needs to be addressed [111]. In one prospective cohort study, a high consumption of cow’s milk at the age of 1 was reported as a dietary risk factor for the induction of β-cell autoantibodies in 2-year-old children [112]. In line with this, the early introduction of cow’s milk, before 5 month of age, was associated with a higher risk for T1D [113]. Overall, cow milk proteins are known to have an intrinsic allergenicity, particularly beta-lactoglobulin, bovine serum albumin, α-casein, and κ-casein [114]. An increased humoral and cellular immunity to cow’s milk proteins in children with T1D is not disease-specific but reflects a genetic predisposition to increased immunity to dietary proteins in general [113]. Thus, an oral tolerance to cow milk antigens could be impaired in individuals with a genetic susceptibility to T1D, and this could trigger autoimmunity [111].

In conclusion, early nutrition could influence traits related to autoimmunity, but these implications need to be better clarified. The role of nutrition later on in life has been investigated only in one study, which showed an association between high sugar consumption and the risk of T1D in adult subjects; meanwhile, one study found that the diet of diabetic subjects might be characterized by a high consumption of UPFs. In view of this, it is not possible to establish whether a pro-inflammatory dietary pattern is a trigger for the development of T1D. Further studies are needed to better understand the mechanisms underlying these associations and whether a pro-inflammatory dietary pattern can worsen a person’s susceptibility to autoimmune diseases like T1D. 

##### Multiple Sclerosis

Multiple sclerosis (MS) is a chronic inflammatory and immune-mediated disease of the central nervous system (CNS), affecting mostly young women, particularly from Europe and North America [115]. MS is characterized by immune dysregulation, which results in the infiltration of the CNS by immune cells, triggering demyelination, axonal damage, and neurodegeneration, and thus development of disability [116]. The pathogenesis of MS is still unclear as it is multifactorial: based on a combination of genetic and environmental factors [117]. Several studies have reported the possible role of dietary patterns as a risk factor for MS and its progression, but the effect of individual dietary components on MS’s pathogenesis has not yet been determined [118,119,120]. 

The indirect role of dietary factors in cardiovascular risk, obesity, or alterations in the lipid profile, and in determining the chronic immune activation and inflammatory status, particularly in the central nervous system due to oxidative stress, is well known [4,119,121,122,123,124,125]. 

Interestingly, a recent observational single-center, cross-sectional study examined the potential association between UPFs’ consumption and MS severity in a group of adult Italian MS patients. Data showed that a higher consumption of UPFs was associated with moderate to high MS severity compared to lower consumption after adjustments for potential background (OR = 2.46, 95% CI: 1.04–5.83) and clinical confounding factors (OR = 2.97, 95% CI: 1.13–7.77) [126]. 

In addition, Fitzgerald et al., in a large cross-sectional survey, showed that a healthy diet rich in fruits, vegetables, legumes, whole grains, and dairy products, and low in added sugars and red meat, and a healthy lifestyle, are associated with a lower burden of disability symptoms in MS, such as depression, pain, fatigue, and cognitive impairment [127]. 

Some studies have highlighted the anti-inflammatory and neuroprotective properties of a Ketogenic diet (KD) in MS [128]. The ketone bodies perform their neuroprotective functions by reducing oxidative stress and ROS production, enhancing NADH oxidation, which inhibits the mitochondrial permeability transition and directly affects the inflammasome [129].

Among the ketone bodies produced by the KD, β-hydroxybutyrate (BHB) reduces L-1β-mediated NLRP3 inflammasome activation [130]. It exerts antidepressant effects, perhaps by inhibiting NLRP3-induced neuroinflammation in the hippocampus [131]. Furthermore, NLRP3 appears to act as a bridge between the innate and adaptive immune responses in the early stages of MS, promoting the migration of macrophages, dendritic cells, and myelin-specific autoreactive CD4+ T cells into the CNS and, therefore, it may be considered a critical factor in the development of neuroinflammation and an interesting therapeutic target in immune-related disorders [132].

Another interesting topic concerns the crosstalk between the gut microbiota and the central nervous system (CNS), called the “gut-brain axis”, which can be bi-directional: “bottom-up” from the gut microbiota to the brain, and “top-down” from the brain to the gut microbiota. Metabolites of the gut microbiota, such as SCFAs, tryptophan (Trp) metabolites, and secondary bile acids, are the key players mediating this bottom-up communication, and, in particular, SCFAs are, which appear to have numerous properties, including neuroactive functions [133,134]. The exact mechanisms by which SCFAs exert their effects are largely unknown but several animal studies have demonstrated their influence on key neurological and behavioral processes that may be involved in neurodevelopmental and neurodegenerative disorders [135]. In addition, the microbiota, by producing neuro-metabolites (such as serotonin/5-hydroxytryptamine, GABA, glutamate, phenylalanine, Trp, tyrosine, carnosine, SCFAs, threonine, alanine, lysine, glycine, serine, aspartic acid, ammonia, and gut hormone/incretins), can influence brain functions and brain influences on gut activity. Therefore, diet plays a role in how the gut–brain axis functions [136].

To conclude, no evidence has shown that diet can be a trigger for the onset of MS. Clinical and experimental studies provide indirect evidence that a balanced diet, combined with an overall healthy lifestyle, is linked to an improvement in several clinical parameters and measures of the quality of life in patients with MS, however, at present, there are no precise recommendations regarding a specific dietary treatment for this disease [118]. Further studies should be conducted to clarify the role of dietary habits in the management of MS.

##### Allergies

The prevalence of atopic diseases, including food allergies, has been increasing in Western countries, becoming a public health problem [137,138]. Food allergies affect up to 10% of the population, and mainly the pediatric one [139]. Allergic symptoms such as asthma, eczema, and wheezing have been associated with an unhealthy diet and a high consumption of UPFs and junk foods [139,140,141,142]. Schütte et al. reported that a pro-inflammatory diet may worsen the atopic outcome and reduce an individual’s capacity to tolerate allergenic molecules [137]. Recent studies have suggested that the nutritional composition of UPFs plays an important role in the development of allergic symptoms [141,142]. Several studies have found a positive association between the consumption of UPFs and allergic symptoms in children and adolescents [141,142,143,144]. Katidi et al. suggested that UPFs may lead to a higher exposure to additives and allergens, which may contribute to the allergy process [145]. This may be due to the higher content of allergens in UPFs compared to less-processed foods [146]. Moreover, individuals with food allergies may develop sensitization to other foods that contain homologous proteins [147]. Therefore, individuals affected by multiple food allergies should be cautious about consuming UPFs [145]. There is increasing evidence that the gut dysbiosis is related to the development of a food allergy, although this specific pathogenesis is still unknown [142,148]. As previously mentioned, UPFs can affect the gut microbiota composition, which in turn might influence the immune system and the risk of developing a food allergy [105] Regarding allergies that are not food-related, the impact of diet on asthma pathogenesis has been investigated [149,150]. A Brazilian retrospective study found a positive association between the highest quintiles of UPFs’ consumption and asthma in adolescents [141]. Regarding specific food components, a high intake of processed meat, defined as >4 servings/week, has been associated with increased asthma symptoms compared to <1 serving/week in a prospective study in adults [140].

Overall, further studies are needed to determine the causal relationship between a pro-inflammatory dietary pattern or UPFs’ consumption and allergic disease [145].

#### 3.1.3. Malignancies 

##### Pediatric Cancer

Although cancer is relatively rare in childhood, it represents a leading cause of death for children and adolescents, with an increasing incidence especially in several developed countries [151,152]. The etiology remains unknown, unlike most adult cancers which are often linked to environmental and lifestyle factors, including diet [153,154].

The impact of dietary patterns on modifying the risk of cancer has been extensively investigated in relation to malignancies occurring in adulthood [153,155,156,157]. However, the role of diet in the development and prevention of cancer in the pediatric population is still unclear. In fact, the rarity of the disease and the challenges associated with assessing early life diet limit the quality and quantity of evidence for causal associations between dietary factors and pediatric tumors [158]. In addition, the malignancies that have been most associated with certain dietary patterns (e.g., the WD and consumption of UPFs), include breast, prostate, and colon–rectal cancers that do not commonly occur in children [155,157]. Furthermore, some of these cancers, such as colon neoplasia, are believed to have latency periods of several decades. In contrast, cancers in children have shorter latencies of no more than 15 years, and often less than 5 years [159].

The study of Harris et al. examined the potential association between an inflammatory dietary pattern during adolescence and early adulthood with the occurrence of breast cancer. Their findings support the hypothesis that a diet characterized by a high intake of sugar-sweetened soft drinks, refined grains, red and processed meat, and a low intake of green leafy vegetables may increase the incidence of premenopausal breast cancer [160]. Even though the consumption of red and processed meat during adulthood has been recognized as a well-established risk factor for the development of colorectal cancer, two large cohort studies found no significant correlation between the early life intake of red and processed meat and the risk of colorectal neoplasia later in life [161,162]. These results highlight the importance of considering the timing of exposure when examining this association [159]. Studies have also evaluated maternal dietary patterns during gestation and the risk for some childhood cancers: a recent systematic review suggested that a high maternal dietary intake of vegetables, fruits, and protein sources, supplemented with folic acid and multivitamins during pregnancy, could have a protective effect against childhood acute leukemia [163].

The current evidence regarding the association between UPFs and cancer risk is still scarce and limited to adult populations [164]. UPFs are indicators of poor food quality and an imbalanced nutritional profile, contributing to weight gain and the risk of obesity, which is recognized as a major risk factor for several malignancies [165]. In addition, UPFs may contain neo-formed contaminants and food additives, for which carcinogenic and endocrine-disrupting properties have been suggested in animal or cellular models [166]. Finally, they have a negative impact on health by altering the gut microbiome, disrupting the energy balance and leading to the growth of microbes that encourage inflammation-based diseases, including cancer [2,167].

On the contrary, the MD is known to have a protective effect, reducing the risk of cancer as highlighted by a systematic review and meta-analysis showing that strict adherence to the MD was related to a lower risk of various cancers as well as cancer mortality [153,168].

To date, there may not be strong evidence to suspect that dietary factors play a significant role in the etiology and prevention of most childhood cancer, as there is in adulthood [169]. However, the rarity of this disease and the time-limited dietary exposure of children could limit the research on this potential association [158]. Indeed, the potential harmful or protective effects of certain diets on cancer risk may be seen in the long-term [159]. Thus, the majority of studies have focused on the more significant role of diet in preventing adult-onset cancer through dietary interventions beginning at young ages [169,170]. 

### 3.2. Dietary Inflammatory Potential in Diseases Requiring Special Diets

#### 3.2.1. Celiac Disease

Celiac disease (CeD) is a chronic immune-mediated enteropathy triggered by the intake of dietary gluten and related proteins in genetically susceptible individuals [171]. It has become one of the most common food-related chronic intestinal diseases among children [172,173] The pathogenesis of CeD is complex, and it still not fully understood. Besides genetic and immunological factors, it appears that other environmental determinants, including dietary patterns, can play a significant role in the development of CeD [174]. 

Auricchio et al. investigated a cohort of children with a genetic predisposition to CeD. The children who eventually developed CeD consumed more carbs and less legumes, vegetables, fruits, and dairy products, suggesting that an increased risk of CeD is not only attributed to the amount of gluten consumed, but also the consumption of pro-inflammatory nutrients commonly found in Western-style diets [175].

Although there is currently a limited understanding of the correlation between the WD and CeD, there are potential causal links indicating that the WD may be involved in CeD pathogenesis by inducing mucosal inflammation, increasing intestinal permeability, and altering the gut microbiota, ultimately leading to endotoxemia [176].

Furthermore, Aguayo-Patrón et al.’s study suggested that an excessive consumption of UPFs could contribute to an increased susceptibility to CeD through a microbiota imbalance. Dietary patterns with high fat and sugar intakes result in intestinal dysbiosis, which promotes a pro-inflammatory immune response and increases gut barrier permeability, allowing gluten peptides to cross the lamina propria and facilitating an immune response in genetically susceptible individuals [12,177,178]. 

At present, the only effective treatment of CeD is a lifelong strict gluten-free diet (GFD) [179]. However, there is conflicting evidence regarding the nutritional quality of a GFD, with some studies indicating its imbalanced nutrient profile due to an excessive intake of sugars and fats and insufficient amounts of carbohydrates, fiber, and micronutrients [180,181].

Additionally, the challenge of following dietary recommendations for individuals with CeD may also be due to inadequate food choices, as they may replace gluten-containing products with highly processed gluten-free alternatives like UPFs [12,182,183,184,185]. Furthermore, Nestares et al. reported that young patients with CeD who consumed more UPFs presented higher levels of oxidative stress biomarkers and pro-inflammatory cytokines compared to CeD children consuming less than 50% of their daily energy intake from UPFs and control children [182]. This can lead to the exacerbation of mucosal inflammation and gut microbiota dysbiosis, which can also aggravate CeD pathophysiology and generate a vicious cycle [17,182,186].

Overall, it is essential to improve nutritional education among individuals with CeD, in order to promote a healthy, balanced dietary regimen starting from an early age [180]. One of the dietary patterns with robust evidence of its beneficial effect on a person’s health status is the MD [187]. The protective effect against CeD may be explained by its anti-inflammatory potential, also partially mediated by an interplay with the gut microbiota [12]. Furthermore, according to a study by Barroso et al., adherence from the first months of life to the MD was associated with lower odds of CeD autoimmunity, suggesting its preventative role in the development of CeD [188]. Therefore, diet seems to plays a key role in the pathogenesis and prevention of CeD [174], albeit with limited evidence in children. 

#### 3.2.2. Phenylketonuria 

Phenylketonuria (PKU) is a rare autosomal recessive inborn error of the metabolism, in which the impaired activity of phenylalanine hydroxylase leads to the accumulation of phenylalanine (Phe) in the blood, which becomes toxic in the brain. Untreated patients present with irreversible intellectual disability, autism, aberrant behavior, developmental problems, microcephaly, motor deficits, seizures, and psychiatric symptoms [189,190]. Treatment is primarily dietetic and based on natural protein restriction combined with the consumption of special low-protein foods and Phe-free L-amino acid supplements (L-AAs) [191]. Although improvements in the administration and composition of protein substitutes have recently been developed, suboptimal outcomes are still reported in the literature, probably due to reduced patient compliance to the diet, but also to their chronic metabolic implications [192,193,194,195]. 

Phe-free L-AAs are protein substitutes with different absorption profiles than intact proteins, due to their higher plasma concentrations, faster absorption peaks, and steeper blood concentration reductions, resulting in their negative impact on the nitrogen balance [196]. Fast absorption is associated with the higher oxidation of amino acids (AAs), higher blood urea nitrogen (BUN) levels, and lower protein accretion than the slow digestion of proteins, which instead ensures the efficient postprandial utilization of dietary nitrogen [197,198]. A high BUN suggests that supplemented AAs are not used for protein synthesis but are instead oxidized for energy production [199,200]. 

BUN is also a marker of proteolysis [198] and muscle is the main organ affected by the catabolic state generated by proteolysis in PKU patients [31]. 

Modifying the Phe-free L-AAs’ absorption kinetics may improve the efficiency of protein metabolism, reduce AAs’ oxidation, and have a positive impact on the nitrogen balance. Prolonging AAs’ uptake may also reduce the incidence and severity of catabolic episodes, thereby reducing fluctuations in blood Phe levels [193] This hypothesis is supported by studies in which the percentage of lean mass is associated with the amount of intact protein intake in the PKU diet [201]. Furthermore, children taking bioactive casein Glycomacropeptide (GMP), a natural peptide almost devoid of Phe, have been shown to have a trend of higher height z scores, a greater lean body mass, and a lower fat mass compared to children who took only AAs or a combination of GMP and AAs [202]. 

Results from a recent study conducted on a murine model suggests that the intake of a Phe-free L-AAs mixture engineered to prolong AAs’ absorption via Physiomimic Technology (AAs-PT) may reduce AAs’ oxidation and, consequently, BUN, by maintaining a good catabolic/anabolic balance. In addition, AAs-PT may reduce muscle degradation and fluctuations in insulin and glucose values, contributing to the maintenance of a normal satiety response [203]. Similar effects on the nitrogen balance and glycemic and insulinemic profile have been observed in healthy adult humans [204]. In conclusion, several protein substitutes are available, but no single one is able to totally mimic natural protein intakes in terms of their impact on the nitrogen balance and metabolic status [192]. 

Another important aspect to consider in patients with PKU is the gut microbiota [205,206]. Even though the disease itself could be directly responsible for the altered composition of the microbiota [190], diet is one of the most crucial determinants of microbiota composition. The quality of the consumed carbohydrates (special low-protein products with added glucose and sugar to ameliorate palatability) is a key factor in determining the gut microbial composition and the production of SCFAs, because of the carbohydrates’ higher daily glycemic index and glycemic load. Indeed, if compared to patients with mild hyperphenylalaninemia (MHP) on a free diet, PKU children adhering to a low-Phe diet have a lower microbial diversity and reduced total SCFA and butyrate content, due to the reduction of certain beneficial species, in particular Faecalibacterium prausnitzii and Roseburia spp., which are the main producers of butyrate [207]; PKU subjects are also depleted of Ruminococcaceae and Veillonellaceae families, while they are enriched in the genera Blautia and Clostridium spp. (family Lachnospiraceae) [208]. This pattern may promote an intestinal inflammatory status [35].

A Brazilian case–control study showed a lower alpha diversity in PKU children’s gut microbiota, although with an enrichment in Prevotella, Akkermansia, and Peptostreptococcaceae. At the metagenome level, prediction of the microbial function suggested significant differences in the starch/glucose and amino acid metabolism of bacterial functions in PKU patients [209]. 

Microbiota alterations related to the use of low-protein products and protein substitutes have also been demonstrated, while starches high in amylose and soluble fibers could increase SCFAs’ production [210]. 

An altered “gut-liver” crosstalk network (“gut-liver axis”) can lead to endotoxemia, increased oxidative stress, and a related risk of NCDs [206]. An increase in the triglyceride-glucose index (TyG index), a marker of low-grade inflammation and peripheral insulin resistance, has also been observed in children with PKU, compared to age- and sex-matched healthy controls. Indeed, Moretti et al. showed a positive correlation between the TyG index and the glycemic load in PKU, which is higher than the normal values, strengthening the hypothesis of a possible link between the quality of carbohydrates consumed and a susceptibility to the development of metabolic disorders [211]. 

In conclusion, a disease-specific diet for patients with PKU, including low-protein products and protein substitutes, with added glucose and sugar to ameliorate palatability, may impair the gut microbiota, inducing a proinflammatory status. Achieving eubiosis by improving the quality of dietary products and blends, and the use of pre-, pro-, and postbiotics, could be both a preventive and therapeutic strategy in this complex disease. In PKU children, the consumption of GMP seems to provide a positive effect on the microbiota’s composition by increasing beneficial bacteria such as Agathobacter and Subdoligranulum, and seems to have prebiotic activity [212].

#### 3.2.3. Glycogen Storage Diseases

Glycogen storage diseases (GSDs) are hereditary metabolic disorders occurring due to the deficiency of an enzyme involved in glycogen metabolism [213]. Hepatic GSDs clinically present with hepatomegaly, a failure to thrive, and fasting intolerance. The most frequently associated laboratory finding is hypoglycemia, which is secondary to the impairment of endogenous glucose production during short fasting [214]. GSD type I (GSDI) is the most frequent and severe form of hepatic GSD [215,216]. Its subtype GSDIb is typically associated with neutrophil disfunction and IBD [217], however, a few cases of IBD have been recently reported in the subtype GSDIa as well [218,219].

Long-term complications in GSDI patients mainly involve the liver, with the development of hepatocellular adenomas (HCA) and hepatocellular carcinomas (HCC); the kidney, evolving into renal insufficiency; growth; joints, with gout manifestations; and bones, with osteoporosis [220,221]. 

For hepatic GSDs, the dietary treatment consists of avoiding fasting by eating frequent meals and eating complex carbohydrates, specifically uncooked cornstarch (UCCS), to combat the impairment of the glycogenolytic pathway and hypoglycemia [222,223].According to latest guidelines on GSDI patients, calories should be provided with 60–70% from carbohydrates, as reported in the last guidelines, however, the intake of complex carbohydrates should not result in overfeeding, which can lead to hyperinsulinemia, insulin resistance, obesity, and nutrient deficiencies [224]. The administration of UCCS should be personalized, based on the patient’s needs, while monitoring their glycemic and metabolic parameters [224]. Overloading UCCS in the diet of these patients may cause dysbiosis, which can promote an inflammatory status both locally in the gut mucosa and systemically. 

The actual etiopathogenesis of dysbiosis in patients with GSDI still remains not fully understood, but this typical dietary pattern may contribute to it influencing the clinical “enterophenotype” [225,226,227]. Indeed, an UCCS overload may lower the fecal pH and decrease the conversion of unabsorbable starches to SCFAs [228]. SCFA-producing bacteria—Coprococcus, Blautia, Anaerostipes, Odoribacter, and Faecalibacterium—were decreased in GSD patients treated with UCCS in comparison to controls [223]. Consistently with previous studies [229], diet was revealed to be the major driver of the difference in the gut microbiome composition between patients and healthy controls [223]. 

Ceccarani at al. (2020) conducted a similar study on GSDI patients, reporting that UCCS intake was associated with Veillonella, Citrobacter, and Akkermansia genera, and negatively correlated to Coprococcus and Clostridium genera [230]. A strong reduction in intestinal microbiota richness and diversity was observed in GSD patients, with a dramatic increase in the phylum Proteobacteria and a relative abundance of Escherichia coli, observed both in type Ia and Ib GSD patients [230]. 

Other microbiota characteristics found in GSD patients potentially related to both diet and the gut microbiota, which may contribute to inflammation, are synthesized in Table 3. 

In addition, a high carbohydrate diet, in association with a high fat intake, seems to promote metabolic stress in the hepatic cells, accelerating carcinogenesis, which is one of the main complications in hepatic GSDs [236]. Gjorgjieva et al. (2018) demonstrated a highly accelerated tumor initiation and malignant transformation into HCC in GSDIa murine models fed a high-fat and high-sucrose (HF/HS) diet in comparison with GSDIa mice and healthy mice fed a standard chow diet [237]. The HF/HS diet exacerbated lipid accumulation and greatly increased the incidence of hepatic tumors and their transformation into HCCs in GSDIa mice, but it did not induce tumorigenesis in wild-type mice. Therefore, the authors concluded that the metabolic reprogramming induced by the G6Pase loss seems to provide a more hospitable environment for hepatic tumorigenesis and that the HF/HS diet probably further accelerates tumorigenesis by providing more substrates for glycolysis and lipid synthesis. Microbiota may contribute to hepatic tumorigenesis [219,220,238]. Indeed, Faecalibacterium was found to have a significantly low abundance in patients with NAFLD, independently of their body mass index and insulin resistance [151]. Although NAFLD may cause liver cirrhosis, which predisposes the liver to HCC, it has been demonstrated that HCC can also develop in the absence of cirrhosis [221,223].

In conclusion, as reported for PKU, these studies showed how profoundly these patients’ disease-specific diet may impair the gut microbiota, and the hepatic cells’ metabolism, inducing a chronic inflammatory status, also systemically, which may contribute to the development of long-term complications in GSD patients.

**Figure 3:**
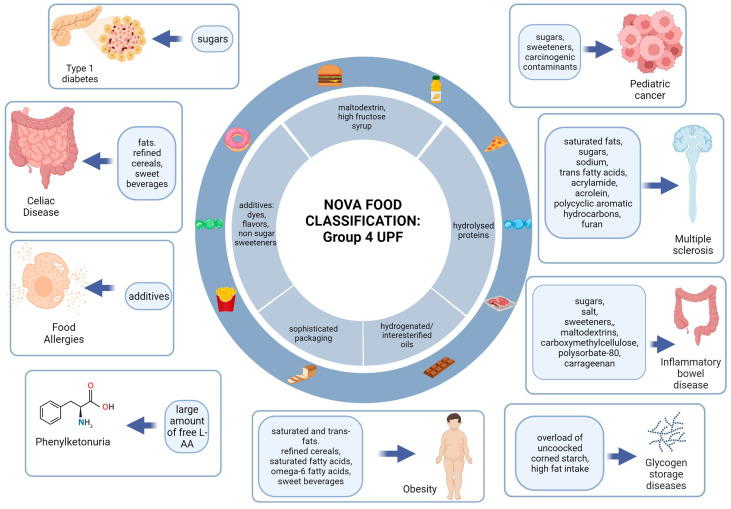
Graphical representation of the main pro-inflammatory nutrients or foods for each disease or group of diseases analyzed.

Figure 3 shows a graphical representation of the main foods or nutrients with pro-inflammatory effects for each disease or group of diseases analyzed. 

The nutritional considerations in Table 4 include some of the main limitations of the studies available in the literature to date. In fact, for obesity, a widespread condition for which more research is available, studies should better detail both the quality of the foods consumed, their content in terms of nutritional composition, and how much the actual intake exceeds or is lower than the dietary guidelines for sex and age. For diseases requiring a specific diet, there are few and heterogeneous studies available for children. Future research should focus more on the specific nutritional changes required to enable disease control, both in terms of dietary patterns and specific nutrients’ intake, and how these may influence the risk of occurrence of other diseases or the composition of the gut microbiota.

## 4. Conclusions

Based on the evidence collected in this literature review, the importance of educating young children and adolescents affected by specific diseases to not only exclude or limit certain types of foods for long-life diet therapeutic reasons, but also to provide quality alternatives and avoid the high consumption of “allowed” but unhealthy foods, emerges. Health professionals must therefore take a wider point of view, consider the entire dietary pattern, and not only focus on pathology. 

Considering multifactorial diet-related diseases, the triggering effect of pro-inflammatory diets has been addressed not only for obesity and metabolic syndrome, but also for adult patients with IBD. In the latter, the main connection between diet and health resides in the gut microbiota and its adequate eubiosis. For multiple sclerosis, the role of diet-related oxidative stress is also emerging, with its consequent chronic immune activation and an inflammatory status that impacts the central nervous system. Future studies will elucidate the implication of dietary pattern in children with T1D and pediatric cancer. Regarding disease requiring special diets, studies reveal that consumption of pro-inflammatory foods can coexist in such conditions. Aware of the mandatory life-long diet therapy, it is up to the specialist to recommend targeted nutritional strategies to limit their consumption. 

Considering the results obtained from this narrative review, it emerges that nutritional studies are still mainly focusing on the study of single nutrients or food intake without giving importance to the complexity of all the diet. Future studies should address nutrition through dietary pattern analysis and focus on other diet-related factors, including dietary habits, lifestyle, socio-economic conditions as well as barriers and resources influencing eating behaviors. 

**Figure 4:**
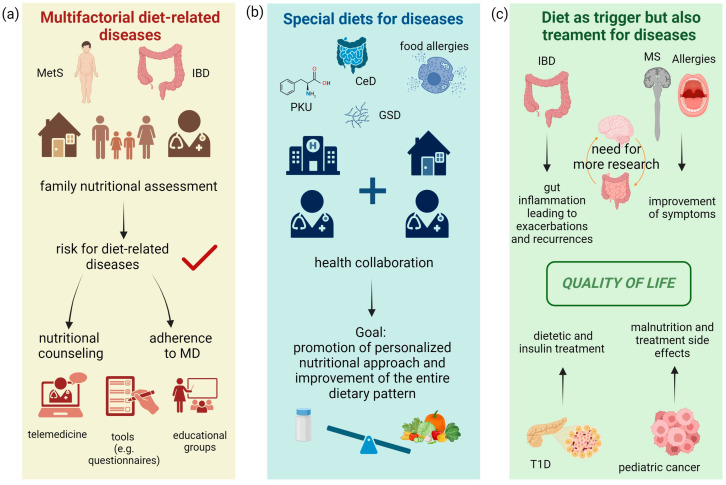
Considerations for diseases’ management and the impact of nutrition. (**a**) The first flow chart shows diet-related diseases. In obesity, there is confirmed evidence in the pediatric setting, and in IBD only in the adult setting. Prevention of these pathologies should start locally, with the family pediatrician evaluating the dietary habits of the family and assessing the presence of risk factors for obesity or IBD. If the risk is confirmed, a tailored intervention to promote the Mediterranean diet should be addressed to the whole family, in collaboration with a nutrition professional, and supporting lifestyle changes with the help of telemedicine tools, educational peer groups, and the administration of Mediterranean diet adherence questionnaires. (**b**) For diseases requiring a special diet (food allergies, CeD, PKU, and GSD), the treatment setting is the hospital, but there must be close collaboration with the family pediatrician. The dietetic nutritional goal is not only the control of the disease itself but the promotion of healthy eating habits through personalized interventions. (**c**) For IBD, multiple sclerosis, T1D, allergies, and cancer, the pro-inflammatory potential of dietary habits during the onset of the disease has been demonstrated in studies on children and adults. After diagnosis, diet also plays an important role in disease management. For IBD, pro-inflammatory dietary habits impact gut inflammation and thus the frequency and intensity of recurrences. There is little evidence for MS and allergies but correct dietary habits could influence symptom control. The gut–brain axis in MS should still be explored. In T1D, the proper management of insulin therapy depends strongly on the patient’s dietary habits. Finally, even for some types of cancer, compliance with nutritional therapy determines the prevention of the risk of malnutrition and the proper management of therapies’ side effects. All these diseases are affected by dietary therapy in terms of the prevention of the recurrence of acute events of the disease, as is the quality of life.

The development of a standard method for detecting dietary pattern adherence is a strategy for considering the diet as a set of factors that simultaneously affect health status. Currently, only some scores to assess children’s adherence to a Mediterranean-like dietary pattern are available, whilst less is known on how to assess pro-inflammatory dietary habits in pediatric age [239]. The implementation of these scores might help in providing targeted nutritional intervention, aimed at reducing the adoption of pro-inflammatory diets. Research should be conducted on the consumption of UPFs in children and identify a standard method (i.e., food frequency questionnaires) to assess their consumption. A final outline of the different implications for the prevention of and medical nutrition therapy for diet-related diseases and for those benefiting from special diets is provided in Figure 4. Finally, in terms of those diseases whose onset is affected by dietary habits, while, at the same time, dietary therapy is an integral part of the treatment of the disease itself, Figure 4 summarizes considerations regarding the importance of nutrition on symptomatology, disease management, and the quality of life for patients. Multidisciplinary management with the active involvement of community professionals, together with the implementation of telemedicine, could not only allow for better dietary adherence, but also reduce the development of pathologies and complications, reducing healthcare costs.

## Figures and Tables

**Table 2:** Dietary food components under investigation in IBD patients and the proposed mechanisms by which they influence the gut microbiota.

Dietary Food Component	Potential Effect	Proposed Mechanism Influencing the Gut Microbiota
Fruit and vegetables	Protective against UC and CD	- ↑ Fermentation of complex CHO with production of SCFAs → preserving the integrity of the intestinal barrier → improving the immune response
Meat	Risk of developing UC and CD	- ↓ oxidation of SCFAs, with alteration of the intestinal mucosa → ↑ permeability to enteric pathogens - Release of carcinogenic and mutagenic molecules from high temperature cooking methods: possible role in exacerbating UC and CD?
Gluten	Frequent gluten sensitivity in IBD patients → substantial uncertainty if gluten-free diet relieves symptoms (under debate)	- Gluten gastro-resistant protein → during digestion ↑ release toxic and antigenic peptides → ↑ damage to enterocytes and their tight junctions → ↓ intestinal barrier
Non-fermented milk and dairy products	Might display a pro-inflammatory effect	- large quantities gastro-resistant proteins, i.e., caseins → potentially implicated in altered intestinal permeability and gut dysbiosis
Fermented milks and dairy products (e.g., yogurt and kefir)	Beneficial effects	- Yogurt consumption has beneficial effects on intestinal function → ↑ numbers of Lactobacillus, Bifidobacterium, and Bacteroides - Kefir has positive impact on IBD symptoms in adults
Salt	Detrimental effect	- ↑ production of pro-inflammatory cytokines and ↑ intestinal permeability → ↓ SCFAs production and ↑ inflammation and in the gut
Artificial sweeteners (e.g., aspartame, saccharin, sucralose, and acesulfame potassium)	Detrimental effect	- can heighten intestinal inflammation by influencing the homeostasis of gut microbiota
Maltodextrins (used to produce soft drinks, candies, and energy products for sports)	Detrimental effect	- ↓ mucus production and impact on intestinal epithelial cells → ↑ intestinal inflammation

Abbreviations: Ulcerative Colitis (UC); Crohn’s disease (CD); carbohydrates (CHO); Short Chain Fatty Acids (SCFA); inflammatory bowel disease (IBD). ↑ = high/greater; ↓ = lower/less; → = as a consequence.

**Table 3:** Microbiota characteristics found in GSD patients which may contribute to an inflammatory status.

Characteristic	Role in Inflammation	References
Depletion of SCFAs-producing bacteria	Reduced anti-inflammatory effect of SCFAs	[223,230]
Enrichment in *Blautia* genus	Induction of cytokines’ secretion by host cells	[230,231]
Depletion of *Firmicutes* phyla	Reduced biodiversity in the human gut	[230]
Depletion of *Oscillospira* and *Faecalibacterium* spp.	Dysbiosis, a constant finding in inflammatory diseases such as Crohn’s disease and nonalcoholic steatohepatitis	[230,232,233]
Depletion of *Christensenella minuta* (*Oscillospira* spp.)	A correlation between Christensenella minuta and a lower BMI has been demonstrated	[230,234,235]
Depletion of *Faecalibacterium*	Association with NAFLD	[221,222]

**Table 4:** Summary of the research and nutritional considerations from the studies collected.

Disease	Studies in Pediatric Age		Research Consideration	Nutritional Consideration
	YES	NO		
Multifactorial diet-related diseases				
Obesity and metabolic syndrome	√		Eight studies on both children and adolescents [43,47,48,50,57,58,64,65]	Nutrition is an environmental factor that influences inflammatory pathways. Several available studies evaluate not only individual nutrients or foods, but investigate nutrition complexity through dietary pattern analysis.
Inflammatory bowel diseases		√	Multiple experimental studies and clinical trials on adult patients	The excessive intake of specific macronutrients enriched in a Western diet affects gut health and promotes gut inflammation.
Immune-mediated diseases				
Type 1 diabetes	√		In two studies early nutrition was evaluated as a trigger for autoimmunity in adolescence and adulthood. With regard to dietary patterns, there is substantial uncertainty. Only association studies on adult patients are available [108,110]	The impacts of the early consumption of cow’s milk proteins, sugar intakes, and UPFs’ consumption was under investigation.
Multiple sclerosis		√	Only one study on adult patients evaluated UPFs’ consumption	UPFs’ consumption in MS should be assessed as early as possible but specific tools are needed.
Allergies	√		Five studies on children [141,142,143,144,145]	UPFs’ intake is associated with allergic outcomes. The role of the microbiota as a mediator between diet and tolerance must be investigated.
Malignancies				
Pediatric cancer	√		Two studies on adolescents [160,162]	UPFs’ consumption should be assessed because of low dietary quality.
Diseases with dietary treatment				
Celiac disease	√		Four studies on children related to UPFs’ consumption [175,180,182,183]	Gluten-free food should be included in the categorization of UPFs.
Phenylketonuria	√		Five studies on both children and adolescents [202,207,208,211,212]	The intake of low-protein products and protein substitutes, with added glucose and sugar to ameliorate palatability, impair the production of SCFAs, because of their higher daily glycemic index and glycemic load. This condition could promote a pro-inflammatory status. Future clinical studies are needed.
Glycogen storage diseases	√		Three studies on both children and adolescents [226,228,230]	The high carbohydrate diet and the overload of uncooked cornstarch (UCCS), which is the main treatment in hepatic GSDs, may impair the gut microbiota and hepatic cells’ metabolism, inducing a chronic local and systemic inflammatory status.

## Data Availability

Not applicable.

## Appendix A. Research Strategies Used in This Narrative Review

1. (“Inflammation”[Mesh] OR “Diet, Western”[Mesh] OR “Food, Processed”[Mesh] OR “Ultra-processed food”[Title/Abstract]) AND (“Pediatric Obesity”[Mesh] OR “Obesity”[Mesh] OR “Metabolic Syndrome”[Mesh]) AND (2008:2023[pdat]) AND (allchild[Filter]). Number of documents = 871
2. (“Inflammation”[Mesh] OR “Diet, Western”[Mesh] OR “Food, Processed”[Mesh] OR “Ultra-processed food”[Title/Abstract] OR “Diet Therapy”[Mesh]) AND (“Diabetes Mellitus, Type 1”[Mesh] OR “Diabetes Type 1”[Title/Abstract]) AND (2008:2023[pdat]) AND (allchild[Filter]). Number of documents = 317
3. (“Inflammation”[Mesh] OR “Diet, Western”[Mesh] OR “Food, Processed”[Mesh] OR “Diet Therapy”[Mesh]) AND (“Inflammatory Bowel Diseases”[Mesh] OR “Colitis, Ulcerative”[Mesh] OR “Crohn Disease”[Mesh]) AND (2008:2023[pdat]) AND (allchild[Filter]). Number of documents = 442
4. (“Inflammation”[Mesh] OR “Diet, Western”[Mesh] OR “Food, Processed”[Mesh] OR “Ultra-processed food”[Title/Abstract] OR “Diet Therapy”[Mesh]) AND (“Multiple Sclerosis”[Mesh]) AND (2008:2023[pdat]) AND (allchild[Filter]). Number of documents = 70
5. (“Inflammation”[Mesh] OR “Diet, Western”[Mesh] OR “Food, Processed”[Mesh] OR “Ultra-processed food”[Title/Abstract]) AND (“cancer*” OR “tumor*” OR ”neoplasia*” [tiab]) AND (2008:2023[pdat]) AND (allchild[Filter]). Number of documents = 26
6. (“Inflammation”[Mesh] OR “Diet, Western”[Mesh] OR “Food, Processed”[Mesh] OR “Ultra-processed food”[Title/Abstract] OR “Diet Therapy”[Mesh] OR “special diet” [tiab]) AND (“allerg*” [tiab] OR “food allerg*” [tiab] OR “Food Hypersensitivity”[Mesh]) AND (2008:2023[pdat]) AND (allchild[Filter]). Number of documents = 223
7. (“Inflammation”[Mesh] OR “Diet, Western”[Mesh] OR “Food, Processed”[Mesh] OR “Ultra-processed food”[Title/Abstract] OR “Diet Therapy”[Mesh] OR “Diet, Gluten-Free”[Mesh] OR “special diet” [tiab]) AND (“Celiac Disease”[Mesh] OR “coeliac disease” [tiab]) AND (2008:2023[pdat]) AND (allchild[Filter]). Number of documents = 958
8. (“Inflammation”[Mesh] OR “Food, Processed”[Mesh] OR “Ultra-processed food”[Title/Abstract] OR “Diet Therapy”[Mesh] OR “special diet” [tiab]) AND (“Phenylketonurias”[Mesh] OR “PKU” [tiab]) AND (2008:2023[pdat]) AND (allchild[Filter]). Number of documents = 64
9. (“Inflammation”[Mesh] OR “Food, Processed”[Mesh] OR “Ultra-processed food”[Title/Abstract] OR “Diet Therapy”[Mesh] OR “special diet” [tiab]) AND (“Glycogen Storage Disease”[Mesh] OR “GSD” [tiab]) AND (2008:2023[pdat]) AND (allchild[Filter]). Number of documents = 18
